# Carfilzomib usage patterns and outcomes in patients with relapsed multiple myeloma: A multi‐institutional report from the Canadian Myeloma Research Group (CMRG) Database

**DOI:** 10.1002/jha2.559

**Published:** 2022-08-31

**Authors:** Arleigh McCurdy, Martha Louzada, Christopher P. Venner, Alissa Visram, Esther Masih‐Khan, Moustafa Kardjadj, Victor H. Jimenez‐Zepeda, Richard LeBlanc, Michael Sebag, Kevin Song, Darrell White, Hira Mian, Julie Stakiw, Anthony Reiman, Muhammad Aslam, Rami Kotb, Engin Gul, Donna Reece

**Affiliations:** ^1^ The Ottawa Hospital Ottawa Ontario Canada; ^2^ London Regional Cancer Center London Ontario Canada; ^3^ Cross Cancer Institute University of Alberta Edmonton Alberta Canada; ^4^ Princess Margaret Cancer Centre Toronto Ontario Canada; ^5^ Canadian Myeloma Research Group Vaughan Ontario Canada; ^6^ Arnie Charbonneau Cancer Institute University of Calgary Calgary Alberta Canada; ^7^ Maisonneuve‐Rosemont Hospital Research Centre University of Montreal Montreal Quebec Canada; ^8^ McGill University Montreal Quebec Canada; ^9^ BC Cancer Agency Vancouver General Hospital Vancouver British Columbia Canada; ^10^ Queen Elizabeth II Health Sciences Centre Dalhousie University Halifax Nova Scotia Canada; ^11^ Juravinski Cancer Center Hamilton Ontario Canada; ^12^ Saskatoon Cancer Centre University of Saskatchewan Saskatoon Saskatchewan Canada; ^13^ Saint John Regional Hospital Saint John New Brunswick Canada; ^14^ Allan Blair Cancer Centre Regina Saskatchewan Canada; ^15^ Cancer Care Manitoba Winnipeg Manitoba Canada

**Keywords:** carfilzomib, multiple myeloma, real‐world data

## Abstract

Carfilzomib is an active and commonly used treatment in patients with multiple myeloma (MM). Using the Canadian Myeloma Research Group Database, we performed a retrospective observational study of patients treated with carfilzomib for relapse of MM in a real‐world setting in Canada between years 2007 and 2020. A total of 445 patients were included in this study: the doublet (Kd/p, *n* = 218) and triplets (KCd, *n* = 88; KRd, *n* = 99; KPd/p, *n* = 40). One hundred and twenty‐two (27%) received carfilzomib‐based treatment in line 2, 133 (30%) in line 3, 90 (20%) in line 4, and 100 (23%) in line 5 or higher. Carfilzomib was dosed weekly in 40% of patients and twice weekly in 60%. The overall response rate of the entire cohort was 57.7%, with 33.6% of patients achieving very good partial response or better. Median progression‐free survival for the overall cohort was 6.3 months with overall survival 19.7 months. This study provides a benchmark for carfilzomib‐based regimens in the real world, demonstrating that these regimens are effective in treating patients with relapsed MM.

## 1 INTRODUCTION

Multiple myeloma (MM) is a hematologic neoplasm characterized by the clonal proliferation of malignant plasma cells within the bone marrow [1]. In the newly diagnosed setting, patients are treated with combination therapy including a proteosome inhibitor (PI) and/or an immunomodulatory drug (IMD), followed by autologous stem‐cell transplantation (ASCT) and maintenance therapy in select “fit” patients. However, MM currently remains incurable; most patients will require therapy for disease at relapse. Several regimens including novel IMiDs, PIs, and monoclonal antibodies (MAbs) are now approved in the relapsed setting leading to improvement in overall patient outcomes [2, 3, 4, 5].

Carfilzomib is a second‐generation PI that irreversibly binds the proteasome. It differs in its structure (tetrapeptide epoxyketone) as compared to the first‐generation PI bortezomib (dipeptide boronate) [6]. Carfilzomib exerts its anti‐myeloma effect via several pathways including unfolded protein stress response induction [7], nuclear factor‐κB activity downregulation [8], increased cell‐mediated MM‐cell lysis [9] as well as via modification of the bone marrow microenvironment [10].

Carfilzomib was initially approved in Canada based on the results of two pivotal phase III randomized controlled trials. In 2015, Stewart et al. [11] published the ASPIRE trial investigating carfilzomib, lenalidomide, and dexamethasone (KRd) compared to lenalidomide and dexamethasone (Rd) in patients with one to three prior lines of therapy. Both progression‐free survival (PFS) (26.3 months vs. 17.6 months) and overall survival (OS) (48.3 months vs. 40.4 months) were improved with the addition of carfilzomib [12]. Subsequently, Dimopoulos et al. [13] completed the ENDEAVOR trial that compared carfilzomib and dexamethasone (Kd) versus bortezomib and dexamethasone (Vd) in patients with one to three prior lines of treatment. Carfilzomib was able to elicit deeper and more sustained responses for PFS (18.7 months vs. 9.4 months) and OS (47.6 months vs. 40.0 months). More recently, the ARROW trial randomized relapsed/refractory MM (RRMM) patients after two to three prior lines to receive carfilzomib weekly (70 mg/m2) versus twice weekly (27 mg/m2). The median PFS was longer in the once weekly group (11.2 months vs. 7.6 months) with a comparable safety profile in this more heavily pre‐treated population [14].

Three additional carfilzomib‐based triplets have been studied. The CANDOR trial studied the combination of daratumumab, carfilzomib, and dexamethasone (DKd) versus Kd, with DKd having a PFS of 28.6 months compared to 15.2 months for Kd [15]. Isatuximab, carfilzomib, and dexamethasone (IKd) was compared to Kd in the IKEMA trial, recently presented with PFS of 35.7 months for IKd and 19.2 months for Kd, respectively [16]. Lastly, the combination of carfilzomib with cyclophosphamide and dexamethasone (KCd) has been studied, with median PFS of 17.2 months reported in a single‐arm phase II trial of patients with one to three prior lines [17], and median PFS of 20.7 months compared to 15.2 months for Kd in a randomized phase trial [18].

There are ongoing studies being conducted with carfilzomib in the front‐line and relapsed setting in both transplant ineligible as well as patients with smoldering myeloma and in many different quadruplet regimens [19, 20]. Carfilzomib is currently not approved in Canada in the newly diagnosed setting or in smoldering myeloma.

There is limited real‐world data with carfilzomib‐based treatments. These data are important in understanding the overall clinical benefits as most real‐world patients would have been excluded from clinical trials due to stringent enrolment criteria [21], with an analysis of six randomized controlled trials (RCTs) revealing that >75% of patients with RRMM in routine practice would have been ineligible for pivotal RCTs [22]. There is some published experience with carfilzomib in single or multi‐center institutions. In a cohort of 293 patients in Europe, it was noted that the overall dosing of patients was lower than published guidelines, which may reflect real‐world dosing for frail or co‐morbid patients [23]. Similarly, in a single‐center retrospective cohort, the cardiac toxicity hypertension was found to be significantly increased compared to historical clinical trial data [24]. Recently, a population‐based analysis from SEER‐Medicare using carfilzomib showed risk factors associated with adverse events including underlying co‐morbidities such as chronic obstructive pulmonary disease or heart failure [25, 26]; however, these population‐based studies are limited by the lack of myeloma‐specific variables including specific regimens as well as dosing and granular toxicity data.

Given the discordance between clinical trial and available real‐world evidence, there is a need to understand the real‐world‐treatment patterns and outcomes of patients treated with regimens containing carfilzomib in RRMM.

## 2 METHODS

We conducted a retrospective observational study using the Canadian Myeloma Research Group Database (CMRG‐DB). This project was approved by the Ottawa Hospital Research Ethics Board in keeping with the CMRG‐DB governance structure. Data were collected from the CMRG‐DB, which is a prospectively maintained web‐based centralized disease‐specific database of over 7000 patients with MM at 15 Canadian institutions, including legacy data from 2007. Local research ethics boards at every contributing site approve entry of patient data into the CMRG‐DB and all patients are consented for research studies. For this analysis, patients were identified that initiated their carfilzomib treatment between July 1, 2009 and December 31, 2020.

Patients were included if they had a diagnosis of MM and were treated with carfilzomib after at least one prior line of therapy. As the CMRG‐DB is a retrospective database on unselected MM patients, some previously enrolled into clinical trials were included in the analysis for this study. Patients were grouped as follows: (1) carfilzomib monotherapy or in combination with corticosteroids (Kd/p), (2) carfilzomib in combination with lenalidomide and dexamethasone (KRd), (3) carfilzomib in combination with cyclophosphamide and dexamethasone (KCd), and (4) carfilzomib in combination with pomalidomide and corticosteroids (KPd/p). Other combinations were not included in the analysis due to insufficient sample size (*n* ≤ 7).

The primary objective of this study was to describe the overall utilization rates of carfilzomib for relapsed MM in second, third, fourth, and fifth or higher lines of therapy. Secondary objectives included determining baseline characteristics of patients initiating carfilzomib as well as determining the dosing of carfilzomib (weekly or twice weekly administration), overall response rates (ORR) by International Myeloma Working Group criteria, PFS and OS of patients treated with carfilzomib and reasons for treatment discontinuation.

PFS is defined as the length of time between the date of first dose of carfilzomib‐based treatment and the earliest date of disease progression or death due to any cause. OS is defined as the length of time between the date of first dose of carfilzomib‐based therapy and death due to any cause.

### 2.1 Statistical analysis

Descriptive statistics were used to report standard baseline characteristics at diagnosis of all MM patients in the CMRG‐DB. Categorical variables were summarized with counts and percentages. Continuous variables were summarized with means, standard deviations, medians, and/or ranges as appropriate.

Time to event analyses were used to assess the PFS and OS curves that were constructed according to the Kaplan–Meier method and the impact of covariates of interest was assessed using the log‐rank test. The summary from Kaplan–Meier analyses included the median and 95% confidence interval of the estimates for each time‐to‐event outcome analyzed. Statistical analyses were performed using R core team 2020 (R‐4.1.1), Vienna, Austria, and RStudio team 2019 (RStudio‐1.4.1717), Boston, MA, USA for Windows. All tests were two‐sided, *p* < 0.05 were considered to indicate a statistically significant result.

## 3 RESULTS

### 3.1 Baseline characteristics

**FIGURE 1:**
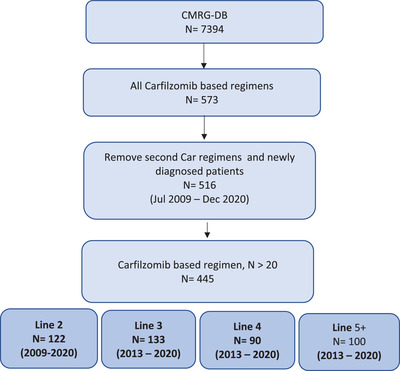
Flow diagram showing selection of study patients between July 2009 and December 2020. CMRG‐DB, Canadian Myeloma Research Group Database

Five hundred and sixteen unique patients were treated with carfilzomib in the CMRG‐DB, of which 445 were identified meeting study criteria (Figure 1). Baseline demographics and disease characteristics by line of therapy are presented in Table 1. At MM diagnosis, median age for the entire cohort was 65 years (range 34–87 years) and 262 (59%) were male. International Staging System stages I, II, and III were present in 29%, 33%, and 38% of patients, respectively, with 23% having high‐risk cytogenetics (deletion 17p, *t*(4;14), or *t*(14;16)).

**TABLE 1:** Patient characteristics at diagnosis by lines of treatment (*N* = 445)

Characteristic	Overall cohort (*N* = 445)	2nd line (*N* = 122)	3rd line (*N* = 133)	4th line (*N* = 90)	5+ lines (*N* = 100)
Age at carfilzomib therapy initiation, median (range)	65 (34–87)	64 (38–82)	65 (34–84)	65 (44–87)	64 (40–85)
Sex, *n* (%) (male)	262 (58.9)	66 (54.1)	78 (58.6)	52 (57.8)	66 (66.0)
MM subtype, *n* (%)^a^					
IgG	231 (54.2)	70 (60.9)	66 (50.8)	38 (43.7)	57 (60.6)
IgA	103 (24.2)	21 (18.3)	37 (28.5)	26 (29.9)	19 (20.2)
FLC	85 (20.0)	23 (20.0)	25 (19.2)	20 (23.0)	17 (18.1)
Other	7 (1.6)	1 (0.8)	2 (1.5)	3 (3.4)	1 (1.1)
Unknown	19	7	3	3	6
ISS stage, *n* (%)^a^					
I	111 (28.9)	33 (30.8)	29 (24.4)	23 (28.4)	26 (33.7)
II	128 (33.3)	31 (29.0)	38 (31.9)	35 (43.2)	24 (31.2)
III	145 (37.8)	43 (40.2)	52 (43.7)	23 (28.4)	27 (35.1)
Unknown	61	15	14	9	23
High‐risk cytogenetics^b^, *n* (%)					
Present	102 (22.9)	33 (27.1)	33 (24.8)	24 (26.7)	12 (12.0))
Not present	142 (31.9)	36 (29.5)	44 (33.1)	23 (25.5)	39 (39.0)
Unknown	201 (45.2)	53 (43.4)	56 (42.1)	43 (47.8)	49 (49.0)

Abbreviations: FLC, free light chain; Ig, immunoglobulin; ISS, International Staging System; MM, multiple myeloma.

^a^ Percentages based on evaluable patients.

^b^ High‐risk cytogenetics defined as deletion 17p, *t*(4;14), and/or *t*(14;16).

### 3.2 Treatment details

Treatment details by line of therapy are shown in Table 2. One hundred and twenty‐two (27%) patients received carfilzomib‐based treatment in line 2, 133 (30%) in line 3, 90 (20%) in line 4, and 100 (23%) in line 5 or higher. Twenty‐four percent of patients received carfilzomib as part of a clinical trial, highest in the KCd cohort at 58%. The majority of patients were PI (94.4%) and IMiD (63.6%) exposed, and the majority had ASCT previously (71.7%).

**TABLE 2:** Carfilzomib treatment details by regimen

Treatment details	All regimens (*N* = 445)	K/Kd/Kp (*N* = 218)	KCd (*N* = 88)	KRd (*N* = 99)	KPd/KPp (*N* = 40)
Line of treatment, *n* (%)					
Line 2	122 (27.4)	25 (11.5)	23 (26.1)	71 (71.7)	3 (7.5)
Line 3	133 (29.9)	68 (31.2)	31 (35.2)	20 (20.2)	14 (35.0)
Line 4	90 (20.2)	54 (24.8)	20 (25.0)	5 (5.1)	11 (27.5)
Line 5+	100 (22.5)	71 (32.6)	14 (15.9)	3 (3.0)	12 (30.0)
Clinical trial, *n* (%)	105 (23.6)	37 (17.0)	51 (58.0)	8 (8.1)	9 (22.5)
Carfilzomib dosing, *n* (%)					
Twice weekly	249 (56.0)	128 (62.4)	20 (23.5)	69 (79.3)	32 (80.0)
Weekly	168 (37.8)	77 (37.6)	65 (76.5)	18 (20.7)	8 (20.0)
Missing	28	13	3	12	0
Prior PI	420 (94.4)	207 (95.0)	79 (89.8)	96 (97.0)	38 (95.0)
Prior IMiD	283 (63.6)	170 (78.0)	57 (65.0)	22 (22.2)	34 (85.0)
Prior ASCT	319 (71.7)	157 (72.0)	68 (77.3)	65 (65.7)	29 (72.5)

Abbreviations: ASCT, autologous stem‐cell transplantation; IMiD, immunomodulatory drug; PI, proteosome inhibitor.

The most commonly used regimens at first relapse were KRd, Kd/p, and KCd used in 58.2%, 20.1%, and 18.9%, respectively. At second relapse, Kd/p became most commonly used at 51.1%, followed by KCd (23.3%), KRd (15.0%), and KPd/p (10.5%). Kd/p was again the dominant regimen used in fourth line (60%) and fifth or higher lines (71%), followed by KCd (22.2% in fourth line and 14.0% in fifth or higher lines) and KPd/p (12.2% in fourth line and 12.0% in fifth or higher lines).

**FIGURE 2:**
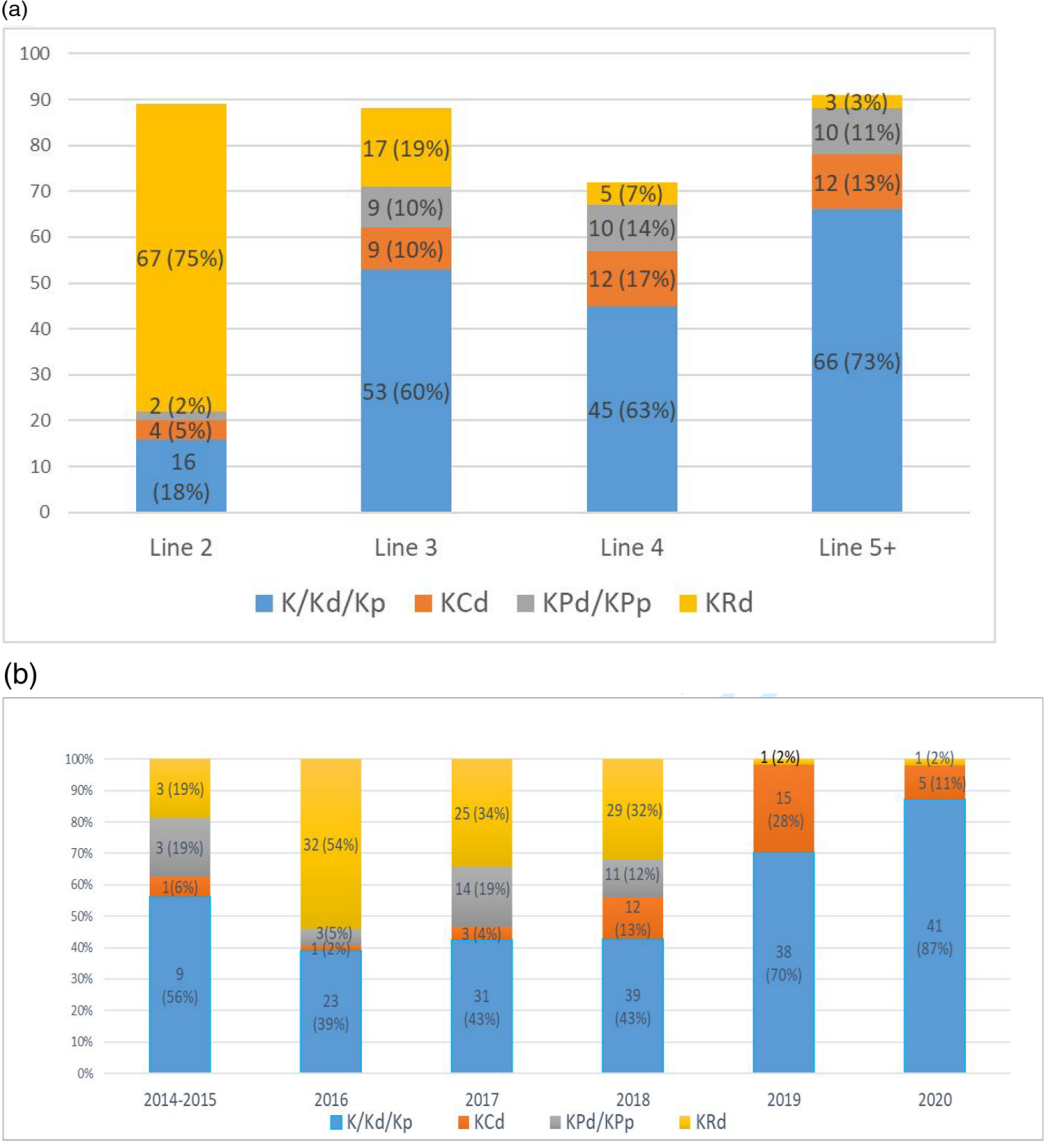
Carfilzomib usage in patients, *n* (percentage) outside clinical trials by (A) lines of treatment and (B) years of study period.

Figure 2A displays regimen use by line of therapy in order to examine regimen prescribing trends at each relapse, while Figure 2B displays regimen use by years during the study period to assess prescribing trends over time throughout the study period. Figure 2A,B excludes patients treated on clinical trial.

In the overall cohort, 249 (56.0%) of evaluable patients were treated with twice weekly dosing and 168 (37.8%) were dosed weekly (Table 2). Twice weekly dosing was more common in all regimens except KCd, of which 65 (76.5%) of patients were dosed weekly. Of note, 51 (58%) of patients treated with KCd were on clinical trial (mostly the Canadian Phase II MCRN003/MYX1 trial [17]).

### 3.3 Response rates, efficacy, and dosing

**FIGURE 3:**
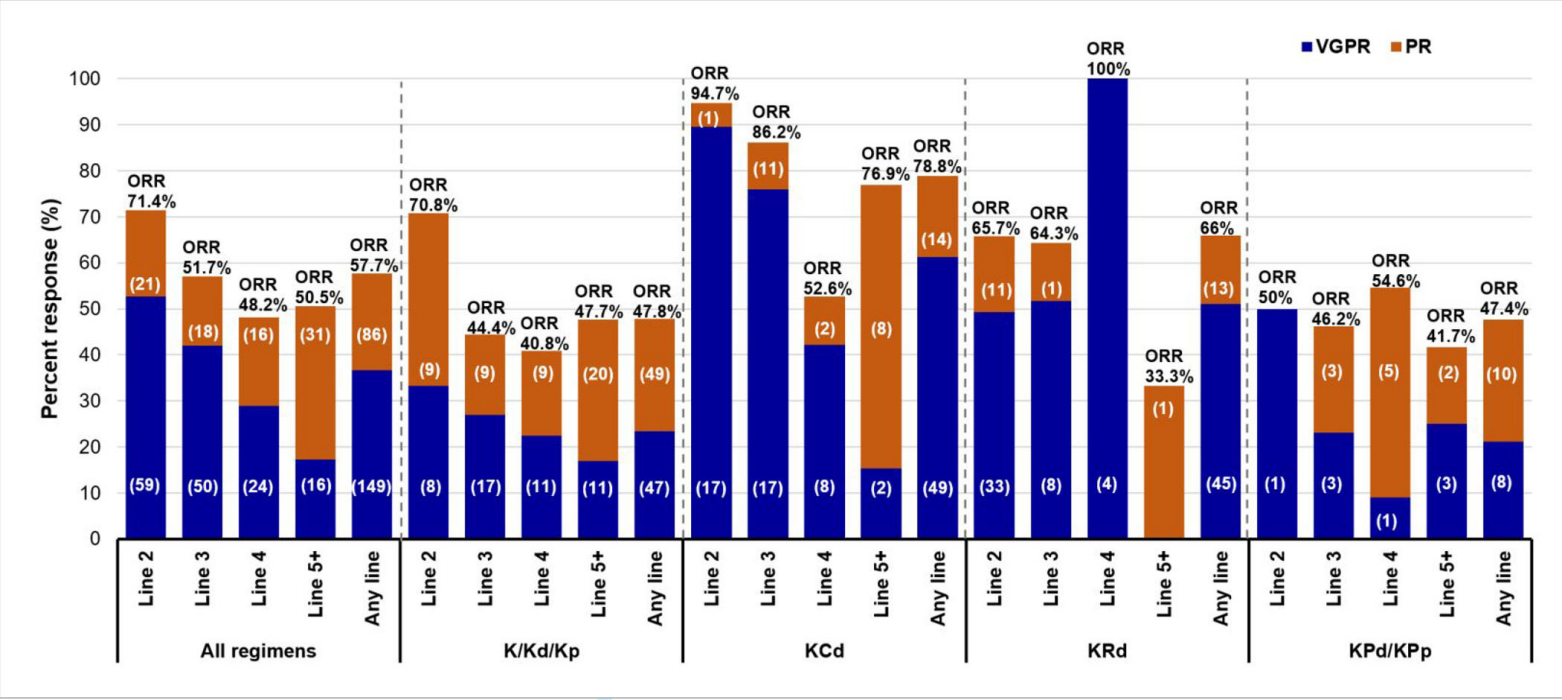
Response rates by regimen and line of treatment. Percentages based on evaluable patients. ORR, overall response rates; PR, partial response; VGPR, very good partial response

Details of response rates by line and regimen are displayed in Figure 3. The ORR of the entire cohort was 57.7%, with 33.6% of patients achieving very good partial response (VGPR) or better. The highest response rates were seen in second line therapy, and this was consistent across all treatment regimens. The triplet combinations of KCd and KRd had higher response rates than Kd/p and KPd/p; however, the KPd/p cohort is much smaller. The ORR remained 50.5% in the 100 patients treated at line 5 or higher, with ≥VGPR rate of 17% in that cohort.

**FIGURE 4:**
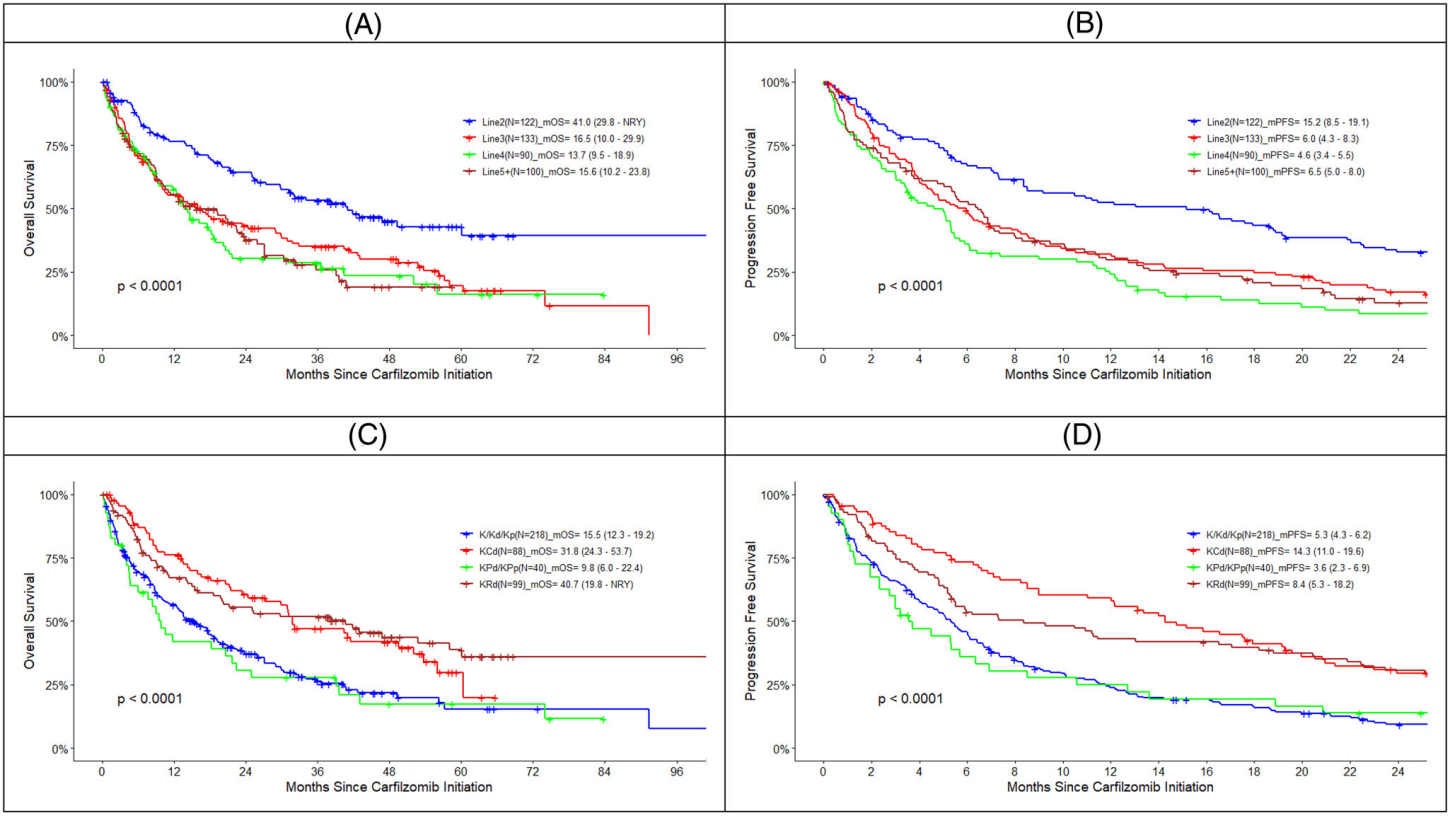
(A–D) Effectiveness by regimen and line of treatment.

Details of PFS and OS by line and regimen are provided in Table 3 and Figure 4. The median PFS for the overall cohort was 6.3 months with OS 19.7 months. The median PFS of 15.1 months and OS of 41.0 months were the longest in line 2, and this was consistent across all regimens. Among all patients by regimen, median PFS in patients treated with Kd/p was 5.3 months, KCd was 14.3 months, KRd was 8.4 months, and KPd/p was 3.6 months. Median OS by regimen was 15.5 months for Kd/p, 31.8 months for KCd, 40.7 months for KRd, and 9.8 months for KPd/p.

**TABLE 3:** Effectiveness by regimen and line of treatment

	All regimens	K/Kd/Kp	KCd	KRd	KPd/KPp
All patients	*N* = 445	*N* = 218	*N* = 88	*N* = 99	*N* = 40
Median PFS	6.3 (5.5–7.4)	5.3 (4.3–6.2)	14.3 (11.0–19.6)	8.4 (5.3–18.2)	3.6 (2.3–6.9)
Median OS	19.7 (76.9–94.0)	15.5 (12.3–19.2)	31.8 (24.3–53.7)	40.7 (19.7–NRY)	9.8 (6.0–22.4)
Line 2	*N* = 122	*N* = 25	*N* = 23	*N* = 71	*N* = 3
Median PFS	15.1 (8.5–19.1)	8.3 (6.0–18.6)	23.9 (17.6–41.0)	11.2 (5.5–23.5)	8.5 (8.5–NRY)
Median OS	41.0 (29.8–NRY)	34.0 (17.7–NRY)	40.0 (31.1–NRY)	41.9 (25.1–NRY)	17.2 (9.4–NRY)
Line 3	*N* = 133	*N* = 68	*N* = 31	*N* = 20	*N* = 14
Median PFS	6.0 (4.3–8.3)	4.3 (3.7–6.1)	13.4 (8.5–41.0)	5.6 (3.9–25.1)	2.8 (2.3–18.9)
Median OS	16.5 (10.0–29.9)	9.9 (7.9–29.0)	30.9 (16.5–NRY)	10.9 (7.2–NRY)	7.9 (4.5–NRY)
Line 4	*N* = 90	*N* = 54	*N* = 20	*N* = 5	*N* = 11
Median PFS	4.6 (3.4–5.5)	3.7 (2.4–5.5)	7.1 (3.0–20.9)	11.2 (5.9–NRY)	3.5 (1.9–NRY)
Median OS	13.7 (9.5–18.2)	13.5 (9.5–18.9)	14.6 (7.9–NRY)	20.2 (14.2–NRY)	8.4 (3.5–NRY)
Line 5+	*N* = 100	*N* = 71	*N* = 14	*N* = 3	*N* = 12
Median PFS	6.5 (5.0–8.0)	6.7 (4.8–9.5)	6.7 (5.3–NRY)	1.6 (0.7–NRY)	4.7 (1.2–NRY)
Median OS	15.6 (10.2–23.8)	13.6 (10.0–23.4)	NR (>13.3)	8.2 (0.7–NRY)	21.4 (4.4–NRY)

Abbreviations: NRY, not reached yet; OS, overall survival; PFS, progression‐free survival.

Forty‐seven patients received carfilzomib‐based therapy specifically when progressing on lenalidomide maintenance post‐ASCT. Seventeen patients received KRd and had an ORR of 92% with median PFS 19.3 months, whereas the 11 patients treated with KCd in this setting had ORR of 73% and median PFS 6.0 months and the 19 patients treated with Kd/P had an ORR of 50% and median PFS 11.2 months.

### 3.4 Toxicity

At the time of the analysis, 414 out of 445 patients had discontinued therapy, while 31 remained on treatment without evidence of progression. Reasons for discontinuation are presented in Table 4. The majority of patients discontinued due to progression or death, which was consistent across all lines. Toxicity was the second‐most common cause for discontinuation, also consistent across all lines.

**TABLE 4:** Reasons for discontinuation by line of treatment

Reason for discontinuation	Overall (*N* = 445)	Line 2 (*N* = 122)	Line 3 (*N* = 133)	Line 4 (*N* = 90)	Line 5+ (*N* = 100)
Progression	295 (66.3)	75 (61.5)	89 (66.9)	58 (64.4)	73 (73.0)
Toxicity	46 (10.3)	13 (10.7)	17 (12.8)	10 (11.1)	6 (6.0)
Death	40 (9.0)	7 (5.7)	13 (9.8)	12 (13.3)	8 (8.0)
Stable disease	31 (7.0)	13 (10.7)	7 (5.3)	7 (7.8)	4 (4.0)
Patient refusal	13 (2.9)	4 (3.3)	4 (3.0)	2 (2.2)	3 (3.0)
No response	8 (1.8)	1 (0.8)	2 (1.5)	1 (1.1)	4 (4.0)
Deepen response	4 (0.9)	4 (3.3)	0 (0.0)	0 (0.0)	0 (0.0)
Lost to follow‐up	4 (0.9)	3 (2.5)	1 (0.8)	0 (0.0)	0 (0.0)
Held due to COVID‐19	3 (0.7)	2 (1.6)	0 (0.0)	0 (0.0)	1 (1.0)
Physician decision	1 (0.2)	0 (0.0)	0 (0.0)	0 (0.0)	1 (1.0)

## 4 DISCUSSION

In this retrospective observational study, we utilized the CMRG‐DB to describe our real‐world experience in Canada with carfilzomib‐based combinations for the treatment of relapsed MM. The results provide insight into which combinations are being used in clinical practice, as well as a benchmark of expected efficacy results for our patients. While our results support the use of carfilzomib in triplet combinations and earlier in the disease course for maximizing outcomes, they also support the use of carfilzomib throughout the disease trajectory, with reasonable response rates and outcomes seen even in patients treated in line 5 and beyond.

Outside of the context of clinical trials and as displayed in Figure 2A,B, during this study period, KRd was the dominant regimen in second line, with Kd/p use increasing in third line and being the most used combination thereafter, which aligns with public drug accessibility at the time. Interestingly, the uses of KRd appears to drop precipitously in 2019, which is when the combinations of daratumumab with lenalidomide and dexamethasone (per the POLLUX trial [27]) or bortezomib and dexamethasone (per the CASTOR trial [28]) became publicly funded and may have been a factor resulting in decreased KRd use.

In our study, the responses and efficacy observed were less than what has been demonstrated in prospective RCTs, but similar to what has been reported in other real‐world series. For example, the ASPIRE trial showed an ORR of 87% [11] with PFS of 26.3 months and OS 48.3 months [12] with a median of two prior lines of therapy compared, whereas the two prior line KRd cohort in our study had an ORR of 64%, median PFS of 5.6 months, and median OS of 10.9 months. Our findings are similar to those reported in other real‐world studies, including an analysis of 208 patients with RRMM treated with KRd after median two prior lines showing a median time to next treatment of 8.6 months, as well as other studies showing similar PFS ranges [29, 30, 31, 32]. The PFS in our study was longest in the KCd cohort in lines 2 and 3. While multiple factors may contribute, it is interesting that this cohort also had the highest use of weekly carfilzomib dosing, administered in 76.5% of patients, which showed superior results compared to weekly dosing in the ARROW trial [14]. Discrepancies in reported outcomes between carfilzomib real‐world data and RCTs could be attributed to suboptimal dosing in the real‐world setting. However, we were, unfortunately, unable to capture dose intensity administered in this cohort, further limiting direct comparisons to randomized studies.

Our study importantly demonstrates that carfilzomib‐based treatments are currently used across many lines of therapy in RRMM, with one‐quarter of Canadian patients who receive carfilzomib doing so in line 5 or higher. Even so, response rates of >50% are maintained among this heavily pre‐treated group, with meaningful PFS and OS outcomes for this selected group of patients. This has been described in other studies, with KCd‐treated patients after median six prior lines showing ORR of 52% and OS 11.9 months in a real‐world analysis [28]. These results support its continued role in this setting, which has been recently challenged in our jurisdiction [33].

Our study has several limitations. We were not able to capture some variables that are not uniformly collected outside of clinical trials and could impact efficacy results in a real‐world setting, including baseline performance status and frailty assessments. In addition, we were unable to describe subsequent post‐carfilzomib treatments in a meaningful fashion, as the heterogeneity of post‐carfilzomib combinations in multiply relapsed was vast, including more than 12 agents in more than 40 unique combinations. Lastly, at the time of this manuscript, carfilzomib is not reimbursed in combination with anti‐CD38 MAbs, though they have received regulatory approvals, which is an important component of modern MM therapy and will require follow‐up in the future to evaluate those outcomes.

In summary, the results of our retrospective CMRG‐DB study provide a benchmark for outcomes with carfilzomib‐based regimens in the real world, demonstrating that these regimens are effective in treating patients with RRMM, with the KRd and KCd combinations used in earlier lines of therapy affording the best outcomes. An improved understanding of the reasons underlying the differences in effectiveness between our results and those reported in clinical trials, including patient factors and the availability of other potent treatments, might lead to improved clinical decision‐making in the future and translate into better real‐world outcomes. Further studies are required to elucidate strategies for optimizing carfilzomib use in the real world and closing the efficacy‐effectiveness gap.

## AUTHOR CONTRIBUTIONS

Arleigh McCurdy, Alissa Visram, Martha Louzada, Christopher P. Venner, Esther Masih‐Khan, and Donna Reece designed research, performed research, collected, analyzed, interpreted data, and wrote the manuscript. Moustafa Kardjadj performed statistical analysis and interpreted data. All authors contributed to data collection, interpreted data, and reviewed the manuscript.

## CONFLICTS OF INTEREST

*Arleigh McCurdy*: honoraria—BMS, Janssen, Amgen, Takeda, Sanofi, and GSK. *Martha Louzada*: honoraria—Janssen, BMS, Amgen, and Pfizer. *Christopher P. Venner*: honoraria—Janssen, Amgen, and Takeda; research funding—BMS and Amgen. *Victor H. Jimenez‐Zepeda*: honoraria—Janssen, Takeda, Merck, and BMS. *Richard LeBlanc*: membership on an entity's Board of Directors or advisory committees—BMS Canada, Janssen Inc., Amgen Canada, Takeda Canada, and Sanofi Canada. *Michael Sebag*: membership on an entity's Board of Directors or advisory committees—Janssen Inc., Amgen Canada, Takeda Canada, and BMS Canada. *Kevin Song*: research funding—BMS; honoraria—BMS, Janssen, Amgen, and Takeda. *Darrell White*: honoraria—Amgen, Antengene, BMS, Janssen, Karyopharm, Sanofi, and Takeda. *Hira Mian*: honoraria—BMS, Janssen, Amgen, Takeda, Sanofi, and GSK; awards—HHS Research Early Career Award from Hamilton Health Sciences Foundation; research funding—Janssen. *Rami Kotb*: research funding—Merck and Sanofi; ownership/share holder—Karyopharm; honoraria—BMS, Janssen, Takeda, Amgen, Sanofi, and Merck. *Donna Reece*: research funding—Otsuka, BMS, Janssen, Takeda, Merck, BMS, and Millennium; consultancy—BMS, Janssen, Amgen, Karyopharm, and Takeda; honoraria—BMS, Janssen, Amgen, Takeda, Sanofi, and GSK. All remaining authors have declared no financial or perceived conflicts of interest.

## ETHICS STATEMENT

The approval for this review was obtained from the Ottawa Hospital Research Ethics Board, Ottawa, Canada.

## PATIENT CONSENT STATEMENT

All patients whose data are reported to the CMRG Database by participating centers have provided informed consent for use of their information for research purposes.

## Data Availability

Canadian Myeloma Research Group Database (CMRG‐DB) governance and research ethics boards at data contributing sites do not allow patient‐level data to be shared. Any aggregate data supporting the findings of this study can be available from the corresponding author upon reasonable request.
